# An Overview of Practical Applications of Protein Disorder Prediction and Drive for Faster, More Accurate Predictions

**DOI:** 10.3390/ijms160715384

**Published:** 2015-07-07

**Authors:** Xin Deng, Jordan Gumm, Suman Karki, Jesse Eickholt, Jianlin Cheng

**Affiliations:** 1 Microsoft Corporation, One Microsoft Way, Redmond, WA 98052, USA; E-Mail: xinde@microsoft.com; 2 Department of Computer Science, Central Michigan University, Mount Pleasant, MI 48859, USA; E-Mails: gumm1jn@cmich.edu (J.G.); karki1s@cmich.edu (S.K.); eickh1jl@cmich.edu (J.E.); 3 Department of Computer Science, University of Missouri, Columbia, MO 65211, USA; 4 Informatics Institute, University of Missouri, Columbia, MO 65211, USA

**Keywords:** protein disorder prediction, applications of disorder prediction, machine learning, deep networks

## Abstract

Protein disordered regions are segments of a protein chain that do not adopt a stable structure. Thus far, a variety of protein disorder prediction methods have been developed and have been widely used, not only in traditional bioinformatics domains, including protein structure prediction, protein structure determination and function annotation, but also in many other biomedical fields. The relationship between intrinsically-disordered proteins and some human diseases has played a significant role in disorder prediction in disease identification and epidemiological investigations. Disordered proteins can also serve as potential targets for drug discovery with an emphasis on the disordered-to-ordered transition in the disordered binding regions, and this has led to substantial research in drug discovery or design based on protein disordered region prediction. Furthermore, protein disorder prediction has also been applied to healthcare by predicting the disease risk of mutations in patients and studying the mechanistic basis of diseases. As the applications of disorder prediction increase, so too does the need to make quick and accurate predictions. To fill this need, we also present a new approach to predict protein residue disorder using wide sequence windows that is applicable on the genomic scale.

## 1. Introduction

Protein disordered regions (also termed intrinsically-disordered regions (IDR)) are segments of a protein chain that do not adopt a stable structure [1,2,3,4]. In some cases, the disordered region can encompass the entire protein, and any protein with an IDR can be referred to as an intrinsically-disordered protein (IDP). Thus far, a variety of protein disorder prediction methods have been developed and have been widely used, not only in traditional bioinformatics domains, including protein structure prediction, protein structure determination and function annotation, but also in many other biomedical fields. The relationship between intrinsically-disordered proteins and some human diseases has played a significant role in disorder prediction for disease identification and epidemiological investigations. Disordered proteins can also serve as potential targets for drug discovery with an emphasis on the disordered-to-ordered transition in the disordered binding regions, and this has led to substantial research in drug discovery or design based on protein disordered region prediction. Furthermore, protein disorder prediction has also been applied to healthcare by predicting the disease risk of mutations in patients and studying the mechanistic basis of diseases. In sum, protein disorder prediction plays an essential role in the bioinformatics field, as well as in other fields, such as biomedicine and healthcare. As a result, protein disorder prediction tools have been widely adopted in various applications, while researchers have been continuously making efforts in improving and developing protein disorder prediction methods.

Given the time and cost associated with identifying protein disorder from experimental methods, a number of computational approaches have been developed to predict protein disorder from a protein’s primary sequence. Many of these computational approaches use machine learning to learn mappings from a protein’s sequence to the ordered/disordered state of a protein [1]. Some examples of this approach include DNdisorder [5], SPINE-D [6], ESpritz [7], RONN [8], DISOPRED [9,10] and PreDisorder [11]. DNdisorder, for example, is a sequence-based approach for predicting disorder developed using ensembles of deep networks. Training of the deep network was done using the DISORDER723 dataset, and the features used as input to the deep network included the position-specific scoring matrix (PSSM), predicted solvent accessibility and secondary structure and some statistical characterizations of amino acid residues. Another sequence-based approach developed using neural networks is SPINE-D, which was developed to predict short and long disordered regions of proteins. It was constructed using a three-layer neural network and one-layer filter for smoothing the predictions. In this method, they trained five independent predictors and averaged results from those predictors, and this was considered as the final prediction. Seven representative physical parameters, a 20-dimension PSSM, predicted secondary structure and predicted torsion-angle fluctuation were used to create the input features for the neural network. ESpritz was designed to predict protein disorder at a faster pace, and it is solely based on protein sequence information, which makes it ideal for annotating entire genomes. This predictor was based on bidirectional recursive neural networks and is an ensemble of three neural networks. The first is for learning the N-terminal sequence context; the second is for learning the primary sequence space; and third is for learning the C-terminal sequence context. Similarly, RONN uses only protein sequence information to make disorder predictions. It is developed using a pattern recognition algorithm called the bio-basis function neural network. In this method, sequences are compared with the series of sequences whose states are known beforehand as ordered, disordered or a mixture of both to calculate an alignment score. Based on those scores, each sequence is classified as ordered or disordered using the neural network model. RONN is unsuitable for recognizing short regions of disorder or the first and last residues of disordered regions. Finally, DISOPRED is another *ab initio* protein disordered region predictor. It was originally trained on high-resolution X-ray crystal structures and uses PSI-BLAST to search over a filtered sequence database to generate sequence information for each protein target. Most of these approaches make use of additional evolutionary information that can be obtained by searching a sequence database. The extra information increases performance, but at the cost of time needed for the search.

In this work, we review a number of applications of *in silico* protein order/disorder prediction. To the best of our knowledge, most previous overview work regarding protein disorder prediction focused on the methodology and performance comparisons of disorder predictors or described how intrinsically-disordered proteins function [1,4,12,13,14,15]. Our review, in contrast, provides a concise overview of specific and successful applications of *in silico* protein order/disorder prediction. There has been an increase in the use of disorder prediction in the past decade, and this is opening up new and exciting avenues for further study. To aid in this line of study, we have developed a new sequence-only prediction method, WiDNdisorder (wide deep network disorder predictor), which makes fast disorder predictions and is applicable to large-scale studies. Our approach differs from existing approaches in that it uses a wide sequence window to leverage more sequence information and makes predictions through a two-stage process. Furthermore, by using deep networks, maxout units and a dropout training procedure, our approach is capable of producing predictions that are on par with state-of-the-art methods in terms of balanced accuracy and Sw score (*i.e.*, roughly the sum of the sensitivity and the specificity), but while making use of much less information (*i.e.*, the input to WiDNdisorder consists solely of the protein’s primary sequence in contrast to DNdisorder and DISOPRED, which use sequence-derived information, such as the output of PSI-BLAST). When comparing our method to other approaches that only use the protein’s primary sequence as input (*i.e.*, those methods designed for large-scale studies and quick genome-wide scanning), WiDNdisorder outpaces their performance in terms of recall for short disorder regions and overall balanced accuracy and AUC.

## 2. Results and Discussion

### 2.1. Review of Practical Applications of Protein Disorder Prediction

#### 2.1.1. Applications of Protein Disorder Prediction in Identifying Biological Evolution and Other Traditional Bioinformatics Domains

Intrinsically-disordered regions (IDRs) fail to adopt a stable structure in their native state. This, however, does not mean that these proteins are necessarily dysfunctional, and indeed, their structural instability and conformational variability have been found to play an essential role in processes such as transcription factor (TF) DNA binding. IDR prediction has aided in the study of these processes and has been useful for investigating many problem domains in traditional bioinformatics, such as biological evolution.

In one study by Huang and Sarai, analysis was carried out on the effect of the mobile flexibility and thermodynamic characteristics of IDPs/IDRs on the evolutionary and functional aspects of proteins [16]. This was done by applying PreDisorder [11] as the protein disorder prediction tool. To supplement the nonsynonymous-to-synonymous substitution rate (dN/dS ratio) as a measure of evolutionary rate, the Shannon entropy of functional motifs in IDRs and ordered regions was calculated and used as an additional measure of variability. The thermodynamic information of experimentally-identified mutations was extracted from the ProTherm database [17]. The result of this work showed that IDRs have a higher evolutionary rate than ordered regions, and the functional motifs in IDPs/IDRs are more conserved than those in ordered regions. Thus, protein disorder prediction can be applied in identifying biological evolution and exploring new functions. A similar study by Chen *et al.* was performed on the impact of alternative splicing and protein structural disorder on mammalian exon evolution by analyzing the dN/dS ratio and the exon type/PIDR (proportion of intrinsically-disordered regions) [18]. DISOPRED [9] and PreDisorder [11] were adopted to predict protein disordered regions in this study, leading to the discovery that IDRs and alternative splicing have independent effects on protein evolution in mammals.

Protein disorder prediction has also been applied to identify some functionally-conserved domains in enzyme and evolutionary domains in protein or DNA binding domains in transcription factors. For instance, protein disorder prediction was used to investigate the prevalence of IDRs in the flanking regions of DNA binding domains (DBDs) in human transcription factors (TFs) [19]. Three distinct disorder prediction tools, PONDR VSL2 [20], DISOPRED2 [9] and PreDisorder [11], were used in this work. It was found that the prevalence of disorder in the flanking regions of DBDs in human TFs is significant and may play an important functional role and potentially influence mutations or natural polymorphisms within exomes.

In addition, protein disorder prediction has been used in some other traditional bioinformatics problems. In a work by Pryor and Wiener [21], the FOLDINDEX webserver [22], ESpritz 1.2 [7], DISOPRED2 [9], SPINE-D [6], PreDisorder [11], VSL2B [20] and seven other tools were used to detect intrinsic disorder in membrane proteins. This work also contains a comprehensive benchmark of disorder prediction methods for identifying disordered regions in membrane proteins.

#### 2.1.2. Applications of Protein Disorder Prediction in Drug Discovery or Design

The flexibility of protein disorder regions makes it possible for a protein to bind to many partners and makes them attractive and novel targets for drug discovery or design. A variety of methods have been proposed for disorder-based rational drug design or discovery [23,24,25,26,27]. Thus, several protein disorder prediction methods have been applied to aid in new drug target discovery or design. One method, called MFSPSSMpred (masked, filtered and smoothed position-specific scoring matrix-based predictor) [28] was proposed to identify MoRFs (molecular recognition features), short disorder-to-order binding regions in disordered protein regions. Anchor [29] and MoRFpred [30] have been utilized in this work to help identifying MoRFs in IDPs, which is an important step in understanding functions of IDPs and designing novel drugs. In another work, it is emphasized that computational tools like DISOPRED [31] can be involved in finding new drug candidates and at a lower cost [32]. Consequently, the application of protein disorder prediction tools promotes drug discovery, which has a profound impact on disease treatment.

#### 2.1.3. Applications of Protein Disorder Prediction in Disease Risk Identification and Other Healthcare Fields

Some studies have shown an intriguing correlation between IDPs and diseases, such as cancer, cardiovascular disease, diabetes and neurodegenerative disease [12,33]. It was discovered that mutations that caused diseases were often located in disordered regions [34]. As a result, protein disorder prediction has been of a great assistance in predicting the disease risk of mutations and studying the mechanistic basis of diseases. In one application by Hu *et al.*, the protein disorder predictor PONDR-VSL2 [20] was used to study the change in the tendency of a protein region to be structured or disordered [35]. The experimental results showed that a significant change in the structural tendency of a protein region may give rise to the malfunction of such a protein and disease risk. In another study, several protein disorder prediction tools, metaPrDOS [36], IUPred [37], DisCon [38], POODLE-S [39] and Anchor [29], were applied to predict the disordered regions of the wild-type and mutant proteins. The *in silico* analysis of disordered regions in the mutations suggested that the increase in intrinsic disorder leads to the protein’s loss of function, which is essential in disease risk identification.

Furthermore, protein disorder prediction has been applied in some other healthcare domains, such as epidemiological investigation. Take one study on the novel coronavirus MERS-CoV as an example [40]. Protein disorder prediction was applied to cluster coronaviruses into three groups, correlated in terms of the levels of oral-fecal and respiratory transmission. MERS-CoV is classified as in disorder group C, in which coronaviruses have relatively hard inner and outer shells, giving rise to the virus’s highest oral-fecal components, but low respiratory transmission components. In this study, disorder prediction of two shell proteins of coronaviruses provides a view into the evolutionary nature of the virus. Hence, disorder prediction can be a powerful tool in further epidemiological investigations and other healthcare problems.

### 2.2. Developing a Scalable Disorder Predictor

Considering the numerous applications of disorder prediction, it would be advantageous if a disorder predictor could make predictions in a timely manner (*i.e.*, a matter of seconds). Such an approach could then easily be applied to studies on the genomic scale. To ensure fast predictions, the input to the method would need to consist of the protein sequence (*i.e.*, sequence only) and would not make use of sequence-derived information obtained, for example through a search of a sequence database using PSI-BLAST. Some sequence-only approaches have been derived and tested (e.g., ESpritz [7]) and are typically not as accurate as those methods that make use of additional sequence-derived information. Here, utilizing recent developments in the machine learning community that produce more robust and generalizable models, a new sequence-only protein disorder predictor is developed and evaluated.

#### 2.2.1. WiDNdisorder: A Fast, Sequence-Based Disorder Predictor

WiDNdisorder (wide deep network disorder predictor) is a sequence-based ordered/disordered predictor suitable for studies on the genomic scale. The method leverages recent advances in machine learning (e.g., deep networks, dropout and maxout) to predict a residue’s state (*i.e*., order/disorder) solely from the residues included in a very wide window centered on the residue under consideration. The approach is a stand-alone method and does not use information stemming from multiple sequence alignment or PSSM. As a result, the method is very fast and capable of making predictions in a matter of seconds.

#### 2.2.2. Evaluation of WiDNdisorder

**Figure 1:**
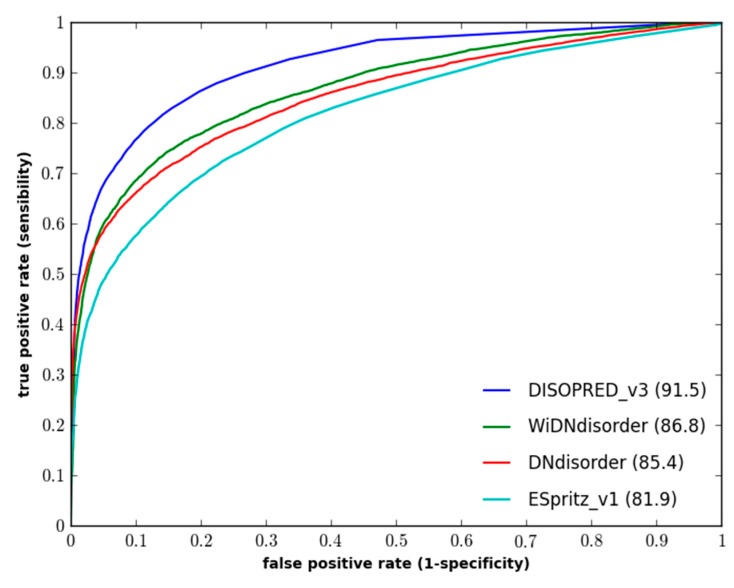
Performance of disorder prediction methods on the DO1111_TEST dataset.

**Figure 2:**
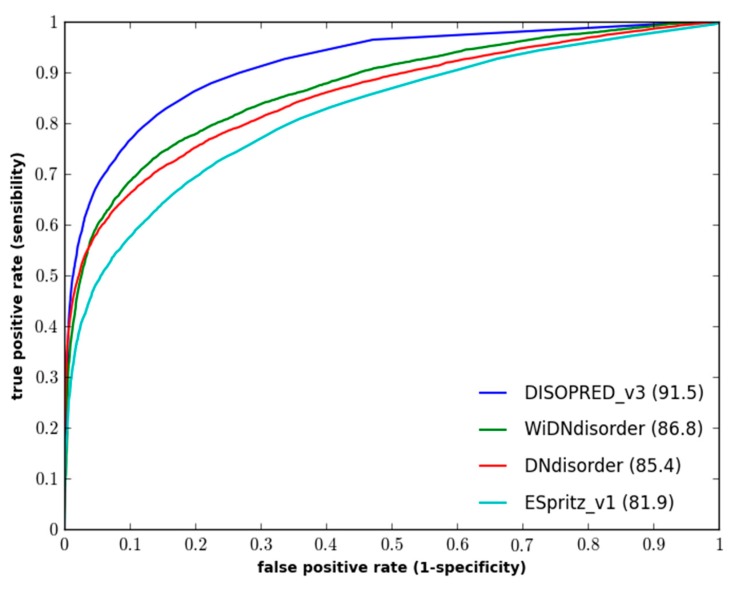
Performance of disordered prediction methods on the CASP10 dataset.

Table 1 and Table 2 show an evaluation of WiDNdisorder with ESpritz [7], DISOPREDV3 [10], and DNdisorder [5]. Of these methods, DNdisorder and DISOPREDV3 make use of additional evolutionary information gathered through a sequence search (e.g., PSI-BLAST [41]). The added evolutionary information provides a modest increase in performance, but comes at the cost of executing the search for homologous proteins. The values are reported for ESpritz, which does not use evolutionary information. DNdisorder was also included in the evaluation, since it is also based on deep networks. The evaluation metrics used include balanced accuracy, F-measure, Sw and area under the ROC curve. These metrics have been used extensively in the literature [5,7,11,42] and used in the official CASP assessments [43,44], and a complete description of the evaluation metrics and associated formulas can be found in Section 3.3. In terms of balanced accuracy (BAC), WiDNdisorder performed quite well on two independent evaluation datasets. With respect to area under the ROC curve (AUC), WiDNdisorder also performed well, outpacing its principle competitor ESpritz and nearing the performance of DISOPREDV3 on the DO1111_TEST dataset. Figure 1 and Figure 2 show the ROC curves for the methods on both evaluation datasets.

**Table 1:** Performance of WiDNdisorder (wide deep network disorder predictor) on the DO1111_TEST dataset.

Predictor	Balanced Accuracy		F-Measure		Sw		AUC	
	Value	±SE	Value	±SE	Value	±SE	Value	±SE
WiDNdisorder	79.6	0.8	67.8	3.9	59.2	1.5	86.8	0.26
ESpritz (v1.3)	73.8	0.4	60.8	1.8	47.5	0.79	81.9	0.30
DISOPRED (v3)	76.8	0.4	68.7	4.7	53.7	0.81	91.5	0.22
DNdisorder	77.6	0.9	67.3	0.7	55.2	1.7	85.4	0.27

**Table 2:** Performance of WiDNdisorder on the CASP10 dataset.

Predictor	Balanced Accuracy		F-Measure		Sw		AUC	
	Value	±SE	Value	±SE	Value	±SE	Value	±SE
WiDNdisorder	71.7	0.8	33.8	1.2	43.3	1.5	80.9	0.63
ESpritz (v1.3)	72.0	0.8	38.7	2.1	43.8	1.6	81.3	0.64
PrDOS-CNF	69.4	0.8	51.2	1.4	38.7	1.7	88.3	0.53
DISOPRED (v3)	69.0	0.9	52.0	1.8	38.0	2.0	87.2	0.55
DNdisorder	73.1	1.0	34.4	0.9	46.2	1.9	82.3	0.62

WiDNdisorder makes order/disorder predictions through a two-stage process. In the first stage (*i.e.*, Tier 1), order/disorder predictions are made for each residue from the protein’s primary sequence, and in the second stage (Tier 2), the initial prediction is refined using the predicted order/disorder state of neighboring residues as additional input to the prediction process. Table 3 shows the results of a comparison of WiDNdisorder (*i.e.*, Tier 2/refined predictions) *versus* the unrefined predictions generated from one wide deep network (*i.e.*, Tier 1 predictions) on both evaluation datasets. This indicates the value of the two-stage prediction approach used by WiDNdisorder. For full details on the distinction between Tier 2 and Tier 1 predictions, consult Section 3.1 and the accompanying figures.

**Table 3:** Comparison of Tier 1 and Tier 2 predictions from WiDNdisorder on the DO1111_TEST and CASP10 datasets.

Dataset	Tier-1		Tier-2	
	AUC	±SE	AUC	±SE
DO1111_TEST	84.1	0.28	86.8	0.26
CASP10	78.8	0.65	80.9	0.63

The performance of WiDNdisorder was also evaluated on disordered regions of various lengths. For this evaluation, only residues from contiguous sets of disordered residues of specified ranges were considered, and the percentage of residues correctly predicted to be disordered was determined (*i.e.*, of the disordered residues in the range considered, what percent were correctly predicted to be disordered). As an example, consider the range 1–5. All order/disorder predictions for residues that pertained to a disorder region in a protein that measured 1–5 residues in length was retained and evaluated. Table 4 and Table 5 contain the results of this evaluation for ranges 1–5, 6–15, 16–25 and >25 on the DO1111_TEST and CASP10 datasets. As only disordered residues are considered in this evaluation, the percentage correctly predicted as disordered corresponds to recall.

**Table 4:** Recall of disordered predictions by disorder region length on the DO1111_TEST dataset.

Predictor	Length of Disordered Region			
	1–5	6–15	16–25	>25
WiDNdisorder	76.2	57.9	67.2	74.3
ESpritz (v1.3)	74.7	63.3	67.4	55.9
DISOPRED (v3)	39.2	43.3	52.2	58.5
DNdisorder	75.4	74.1	76.1	57.9

**Table 5:** Recall of disordered predictions by disorder region length on the CASP10 dataset.

Predictor	Length of Disordered Region			
	1–5	6–15	16–25	>25
WiDNdisorder	57.5	60.0	57.8	50.8
ESpritz (v1.3)	46.2	52.7	65.6	52.9
PrDOS-CNF	28.7	39.8	46.9	46.1
DISOPRED (v3)	25.1	33.6	49.8	47.5
DNdisorder	48.8	64.6	66.0	53.4

As an initial investigation into the benefit of wide input windows and combinations of dropout and maxout nodes for this particular protein prediction task, a number of disorder predictors were trained for input windows of size 31, 51, 71 and 91. The AUC for each combination of window size and network architecture (*i.e.*, dropout only, maxout nodes only, dropout with maxout nodes) was calculated for the CASP10 and D01111_TEST datasets, and the results are shown in Table 6 and Table 7. Larger input window sizes and the combination of dropout and maxout nodes consistently led to better performance.

**Table 6:** AUC for disorder predictions by input window size on the D01111_TEST dataset.

Deep Network Configuration	Input Window Size (in Residues)			
	31	51	71	91
dropout only	78.6	81.6	82.6	83.3
maxout nodes only	78.0	79.2	80.8	80.9
maxout nodes with dropout	81.7	83.3	83.7	84.1

**Table 7:** AUC for disorder predictions by input window size on the CASP10 dataset.

Deep Network Configuration	Input Window Size (in Residues)			
	31	51	71	91
dropout only	71.0	70.0	73.0	74.9
maxout nodes only	71.3	73.0	74.7	75.8
maxout nodes with dropout	76.7	77.7	78.4	78.8

Finally, to evaluate the speed at which WiDNdisorder is capable of executing, the time required to predict the ordered/disordered state of each residue in a protein was calculated using the time command (*i.e.*, a built-in command available in a Linux command line environment that can determine the execution time of a program). A prediction script was created, which took as input a FASTA file for a protein, predicted the ordered/disordered score for each residue in the protein and then saved the results to a file. The time command was used in conjunction with the prediction script to calculate the entire time needed make predictions for each individual protein in the evaluation datasets. The average execution time needed to make ordered/disordered predictions for a protein was 7.86 and 7.19 seconds on the DO1111_TEST and CASP10 datasets, respectively.

### 2.3. Discussion

From Table 3, it is clear that there is benefit in the two-tiered prediction process employed by WiDNdisorder. The first tier makes predictions from the sequence alone and the second tier uses the predicted order/disorder values of the neighboring residues, as well as the original sequence information to make a refined prediction. This results in a relative improvement of ~2% in AUC.

When comparing WiDNdisorder to other methods, it is important to bear in mind that WiDNdisorder was designed to make quick and robust predictions. As a result, it is comprised of 2 DNs and does not use additional sequence-derived evolutionary information (e.g., position-specific scoring matrix, anchored multiple sequence alignment). Both DISOPRED3 and DNdisorder make use of this extra information, and both methods also make use of several predictors (*i.e.*, DNdisorder is a boosted ensemble of 175 DNs and DISOPRED3 is comprised of three complementary methods). This leads to better performance, but the prediction times are much longer, and it would be difficult to apply these approaches at the genomic scale. Still, WiDNdisorder compares well with both DISOPRED3 and DNdisorder in terms of balanced accuracy and Sw on both the CASP10 and DO1111_TEST datasets. In terms of AUC, WiDNdisorder lagged behind DISOPRED3 and PrDOS-CNF (*i.e.*, one of the best methods from CASP10). When considering other approaches, which are not consensus methods nor use evolutionary information, such as ESpritz, WiDNdisorder is quite competitive and outpaced ESpritz on the DO1111_TEST dataset. It is possible that this performance could be further enhanced by combining it with additional deep networks or other fast approaches to create a small ensemble or consensus predictors.

When comparing the methods, it is also important to consider the datasets used to create a particular method. To construct WiDNdisorder, two distinct sources of disordered definitions were used (*i.e.*, DISORDER723 with disorder defined as missing residues from PDB structures and DisProt in which disorder was determined using a variety of experimental techniques). Proteins with longer IDRs may have trouble forming crystalline structures and be difficult to determine experimentally using X-ray crystallography. As a result, disordered datasets that only or mostly contain definitions from these types of structures may not contain adequate examples of longer IDRs or types of IDRs that would lead to problems preventing a protein’s structure to be determined experimentally. By using more than one source and type of IDRs, WiDNdisorder should be more generalizable to all types of IDRs. A similar process was used by Jones and Cozzetto [10] in the construction of DISOPREDV3. For ESpritz, three different methods were developed using three different training sets with IDR definitions stemming from three different sources (*i.e.*, structures determined from X-ray crystallography, structures determined using NMR and DisProt) [7]. In this evaluation, ESpritz predictions were made using the “X-ray” model. As reported in the results, ESpritz had an AUC of 81.9 on the DO1111_TEST set. When considering only the proteins in DO1111_TEST that came from DISORDER723 (*i.e.*, proteins in which the IDRs were determined from X-ray structures), ESpritz achieved an AUC of 86.3. WiDNdisorder achieved an AUC of 82.8 on the same reduced test set. As is to be expected, ESpritz performed better on these targets, since the IDRs are more similar to those in its training set. With WiDNdisorder, the desire was to strike a balance and to create one method from all of the IDR definition types. WiDNdisorder performs quite well on the varied dataset and is competitive with other more tailored methods, such as ESpritz.

In addition to providing fast, quality order/disorder predictions, WiDNdisorder is novel in its application of a wide, fixed width sequence window as an input to the classification method. While other machine learning approaches have made use of the entire protein sequence for a protein structural prediction task (e.g., residue to residue contact prediction with recurrent neural networks), the input to feed forward neural networks has generally been limited to the range of 5–35 residues. Limiting the input to residues neighboring the target residue makes sense conceptually, since many structural features are more of a localized phenomenon. While the global topology of the protein does play a role, encoding more of the protein sequence typically results in poorer performance, as the machine learning method has difficulty distinguishing the limited signal present in the added input. With this work, however, it was found that larger windows (e.g., 91 residues) provided better performance. We hypothesize that the maxout nodes and dropout learning procedure guarded against overfitting or learning the noise in the longer range sequence data that was provided as input. The resulting deep network was then able to make use of the added data for a modest boast in performance. We are planning further studies to more extensively study this point and to investigate if a wider input window along with dropout and maxout nodes would be useful for other protein structure prediction tasks.

## 3. Experimental Section

### 3.1. Construction of WiDNdisorder

**Figure 3:**
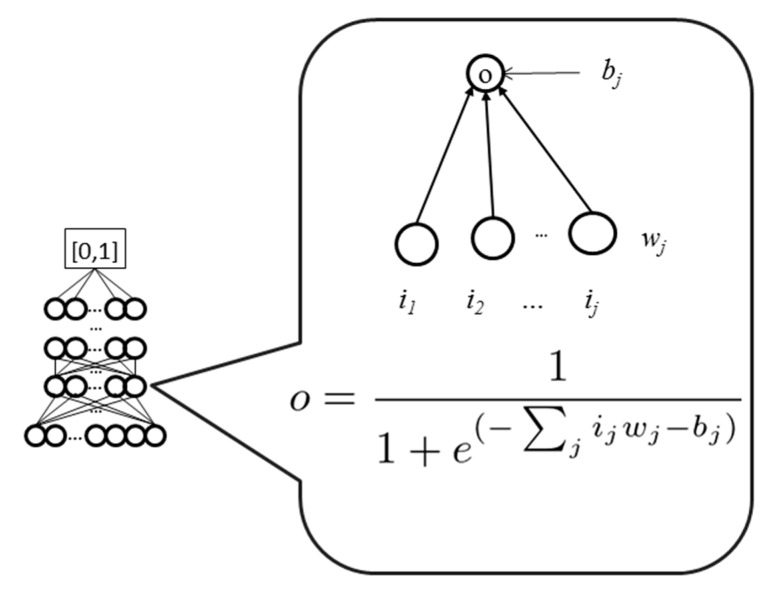
Graphical representation of a neural network.

The underlying architecture of WiDNdisorder is a deep neural network. In simplest terms, a neural network can be viewed as a function that maps a numerical characterization of a phenomena to a particular real value that can be used to represent a class. To increase the expressive power of the neural network, several functions can be composed together in a layered fashion. Figure 3 graphically represents how the functions can be composed and the output of a neural network calculated. The lowest layer represents the input to the neural network. The values of those nodes can then be used to calculate the value of a node in the following layer by multiplying the value of each node by a weight and sending the sum of these products through a transform function (e.g., sigmoid). It is the collection of weights that parameterize the neural network, and through the process of training, they are adjusted. By using a collection of labeled data (*i.e.*, inputs with known classifications), the weights in the neural network can be adjusted such that each input maps to the proper label. When properly trained, the neural network can be applied to predict the labels of novel data. A deep neural network is distinct in that it consists of several layers and as a result can learn more patterns and correlations in the data and use these to increase overall performance.

Intuitively, the ordered/disordered state of a residue is a localized property and will depend to some extent on the ordered/disordered state of neighboring residues. With this in mind, a two-tiered order/disorder predictor was constructed and used to create WiDNdisorder. This idea mirrors the work produced by Spencer *et al.* in which local structural properties of a protein were predicted several times through an iterative prediction process [45]. The advantages of the two-tiered prediction are two-fold. First, it allows for a refinement of the previous prediction; and second, it allows the predicted ordered/disordered state of neighboring residues to be taken into account when making the final prediction for a residue.

**Figure 4:**
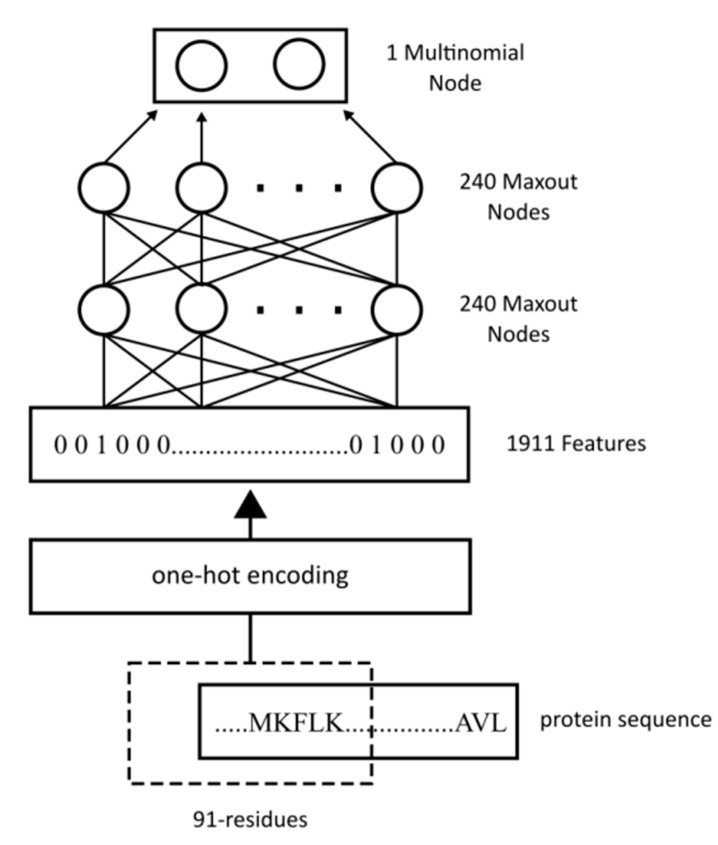
General deep network architecture used for Tier 1 order/disorder prediction.

**Figure 5:**
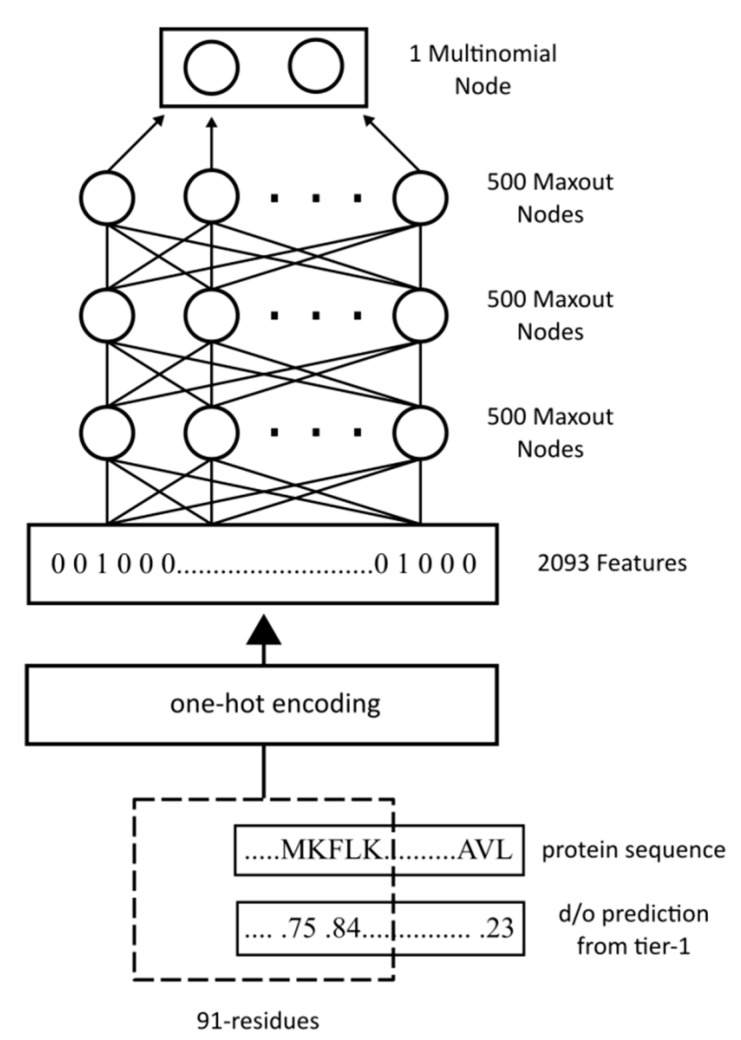
Tier 2 deep network architecture used to make final order/disorder prediction.

With WiDNdisorder, the deep networks consist of four layers. For Tier 1, the input layer consists of 1911 features, and the second and third layers each consist of 240 maxout nodes [46]. The final output is a two-class multinomial node. The deep network was trained using the PyLearn2 library [47] with stochastic gradient descent on batches of 1000 training examples. In addition to the maxout nodes, a dropout procedure [48] was used, which randomly dropped out the output of some of the nodes as their values were propagated through the network. This results in a more robust method less prone to overfitting. To select the hyper-parameters for this tier, a grid search was performed using Jobman. The objective was to maximize the area under the ROC curve on the DO1111_VALIDATION dataset. Figure 4 shows the general architecture of the method for Tier 1. The second stage predictor for WiDNdisorder (*i.e.*, Tier 2) was formed using DO1111_TRAIN and DO1111_VALIDATION, the same training and validation sets used for Tier 1. Since Tier 2 refined the predictions from Tier 1 (*i.e.*, re-predicted the order/disorder state using the initial input and the predicted order/disorder state of neighboring residues), the training set was split into two sets. A model was created for each half of the training set and then used to make predictions for the other half. These predictions were used as additional features for the second-stage predictor. For Tier 2, the input layer consisted of 2093 features. The final predicted value is then sent through a linear transformation to scale the output such that the balanced accuracy was maximized on the validation set when using a threshold of 0.5. Figure 5 illustrates the overall architecture of Tier 2.

### 3.2. Datasets and Feature Generation

The principle dataset for this work was created by merging the DISORDER723 and the DisProt v6.0 datasets. DISORDER723 consists of 723 proteins whose experimental structure was determined by X-ray crystallography and deposited in the Protein Data Bank [49]. Each protein sequence in this dataset is at least 30 residues long, and residues missing from the experimentally-determined structure were considered disordered. This dataset was initially used to develop DISpro [50] and later used to produce PreDisorder [8] and DNdisorder [2]. The DisProt dataset is a set of proteins that contain regions that have been experimentally determined to be ordered or disordered [51]. For this work, Version 6.0 was downloaded from http://www.disprot.org/. It contains of 694 proteins sequences, each of which is a minimum of 27 residues in length. It should be noted that in the DisProt datasets, there were some residues that did not have an experimentally-determined target (*i.e.*, there was no mention of the residue being ordered or disordered). These residues were not used in training or evaluation in order to avoid introducing noise into the training data.

**Figure 6:**
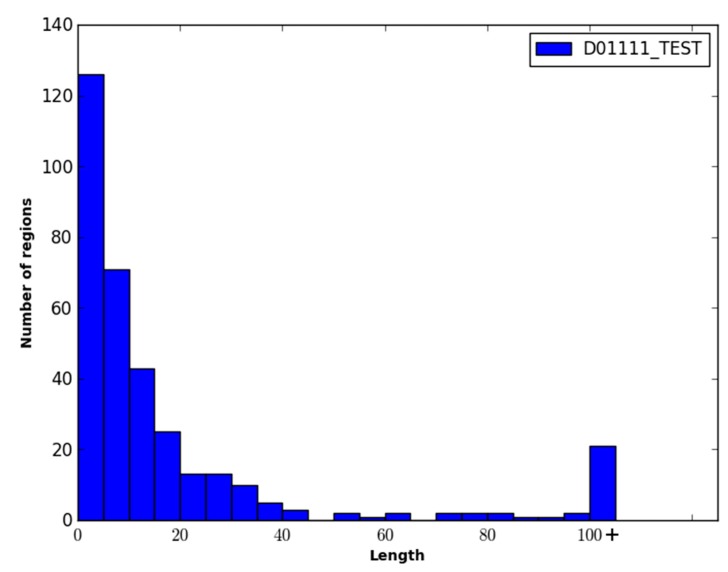
Distribution of the length of disordered regions in the DO1111_TEST dataset. Each bin represents a range of five residues, and the last bin represents the number of disordered regions that have a length greater than 100 residues.

**Figure 7:**
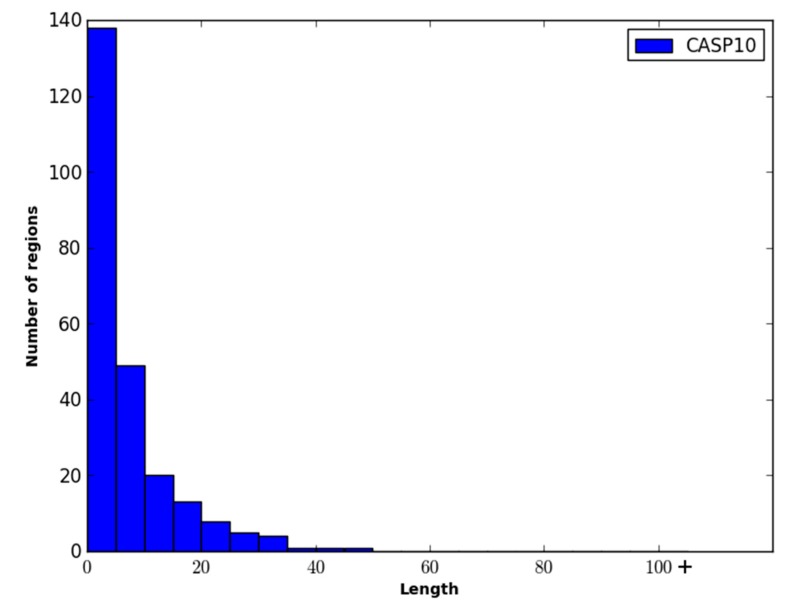
Distribution of the length of disordered regions in the CASP10 dataset. Each bin represents a range of five residues, and the last bin represents the number of disordered regions that have a length greater than 100 residues.

**Figure 8:**
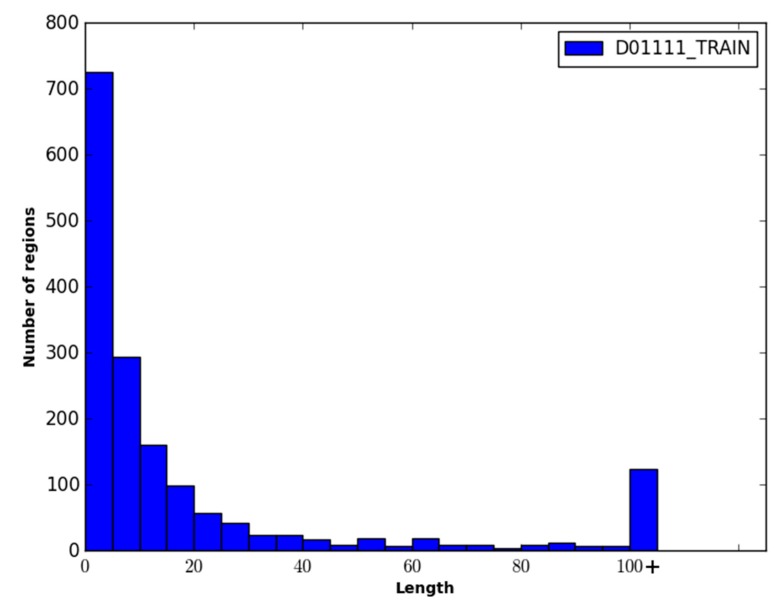
Distribution of the length of disordered regions in the DO1111_TRAIN. Each bin represents a range of five residues, and the last bin represents the number of disordered regions that have a length greater than 100 residues.

The two datasets were combined and filtered such that the pairwise sequence similarity between any two proteins in the dataset was ≤35%. This resulted in the final dataset called DO1111, containing 604 protein sequences from the DISORDER723 dataset and 507 protein sequences from the DisProt dataset. Of these protein sequences, the ratio of order to disorder was approximately 2.2:1 (*i.e.*, ~32% of the residues were disordered). Note that the percentage of disordered residues present in the DO1111 dataset is higher than one might expect (*i.e.*, ~5%–10%), and this is because the majority of the order/disorder annotations in the DisProt dataset are for disordered residues. To facilitate training and evaluating WiDNdisorder, the dataset was randomly divided into three datasets DO1111_TRAIN, DO1111_VALIDATION, DO1111_TEST, with each set comprising ~70%, ~15% and ~15% of the proteins, respectively. This was done to create three independent datasets, and DO1111_TRAIN and D01111_VALIDATION were used exclusively for training and adjusting the learning parameters (e.g., selecting the number layers, the number of nodes per layer, *etc.*). Once the training of WiDNdisorder was complete, D01111_TEST was used for evaluation. D01111_TEST was not used or inspected during the development of WiDNdisorder to ensure fair assessment of WiDNdisorder’s performance. For further evaluation, the CASP10 dataset was downloaded from the official CASP website (Available online: http://www.predictioncenter.rg/casp10/). This dataset contained 95 proteins, among which roughly 6%–7% of the residues were disordered. Figure 6, Figure 7 and Figure 8 show the distribution of the length of the disordered regions in the two evaluation datasets and the training dataset. In terms of the relative percent of disordered regions, the three datasets are similar in nature, with the exception of CASP10, which has fewer IDRs of a length greater than 25 residues.

**Figure 9:**
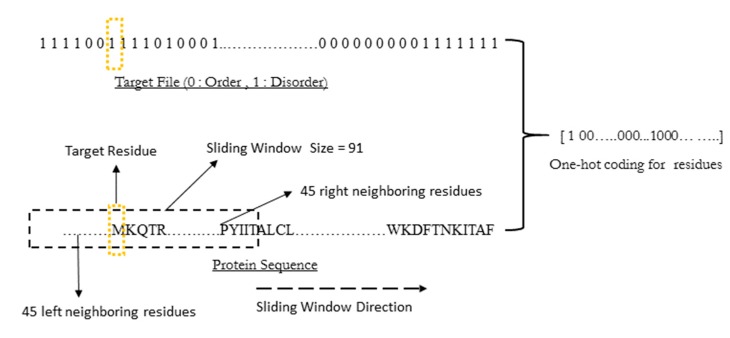
Wide windows used for sequence encoding.

As input to WiDNdisorder, the protein’s primary sequence had to be characterized numerically to be feed into the deep network. The input to a neural network is often referred to as a feature, and with WiDNdisorder, features were encoded to represent the protein sequence for areas surrounding the target residue (*i.e.*, the residue to be predicted as ordered or disordered). These features came from a fix window of 91 residues in length and centered on the target residue. (See Figure 9 for an illustration). For each residue in the window, 21 binary values were used to encode the residue type or missing residue. This provided a means to represent each of the 20 standard amino acid types and a missing residue (e.g., a missing residue would be a position in the fixed window that does not cover the protein’s sequence). This was done using a one-bit hot encoding scheme and, for Tier 1 predictions, resulted in an input feature vector that was 1911 features long. It is important to note that this method only uses the sequence as input and does not make any use of sequence-derived information (*i.e.*, PSSM, multiple sequence alignments or secondary structure prediction). To generate features for Tier 2, the same encoding process was used as previously described for Tier 1 with the addition of two more binary values for each residue in the window. The 22nd feature for each residue in the window was used to encode if a predicted disorder value was encoded (*i.e.*, set to one if a predicted value is encoded), and we used the last bit to represent the real value prediction for a particular residue (stemming from the Tier 1 prediction) or zero if no prediction was available (*i.e.*, if the position in the window did not cover the protein’s sequence). This resulted in a final input vector 2093 long in size for the Tier 2 prediction.

### 3.3. Evaluation Metrics and Data Collection

For the evaluation of WiDNdisorder, area under the ROC (AUC) and balanced accuracy (BAC) were used as the principle metrics. The AUC is the total area under a plot of the true positive rate *versus* the false positive rate across a range of decision thresholds. It provides a measure of a method’s overall effectiveness irrespective of any particular decision threshold. With the AUC, a higher score indicates better performance, and AUC values range from 0.5 for a random classifier to 1.0 for a perfect classifier. The balanced accuracy is the simple arithmetic mean of the positive predictive value (PPV = (TP/(TP + FP))) and the negative predictive value (NPV = (TN/(TN + FN))). Given the skewed nature of the number of ordered to disordered residues in most datasets, the balanced accuracy provides a better sense of a method’s performance on both ordered and disordered residues for a fixed decision threshold. Additionally, the F-measure and Sw score (*i.e.*, (TP/(TP + FN)) + (TN/(TN + FP)) − 1) were calculated. All of these metrics have been used extensively in the literature [5,7,11,42] and used in the official CASP assessments [43,44]. The recall (*i.e.*, TP/(TP + FN)) for disordered predictions was used to assess each method’s performance for both short and long IDRs. TP (or TN) refers to a residue that was predicted to be disordered (or ordered) and was experimentally determined to be in the predicted state. FP and FN refer to incorrect predictions. The values reported in Table 1, Table 2, Table 3, Table 4, Table 5, Table 6 and Table 7 were all scaled by multiplying the calculated value by 100. Approximations for the standard error (SE) were obtained through a bootstrapping procedure in which 80% of ordered/disordered predictions for a method were sampled 1000 times.

In collecting the ordered/disordered predictions, DISOPREDV3, DNdisorder and WiDNdisorder were run locally. For ESpritz, the web service was used to make predictions for both datasets, selecting the “X-ray” dataset and “best Sw threshold”. For PrDOS-CNF, the predictions for the CASP10 targets were downloaded from the official CASP10 data repository (Available online http://www.predictioncenter.org/download_area/CASP10/predictions/). For all methods, the decision threshold used to calculate recall, BAC, Sw and F-measure was 0.5 with the exception of ESpritz, which used a threshold of 0.064.

## 4. Conclusions

In this work, we reviewed the emerging applications of protein disorder predictions in a number of biomedical fields, such as protein evolution, protein function, membrane proteins, drug discovery, drug design, analysis of disease risks and some healthcare domains. The existing work demonstrates that disorder prediction has become an indispensable tool for many research and medical problems in these fields. In order to facilitate the large-scale application of disorder prediction to big data produced in this field, we also developed a new deep learning method to predict protein disorder from one single primary sequence only, which is several magnitudes faster than existing methods and has comparable performance. The method can be a valuable tool for large-scale scanning of big genomics data for identifying disorder proteins that are relevant to many biomedical problems.
